# Community acquired fungemia caused by *Candida pulcherrima*: diagnostic contribution of MALDI-TOF mass spectrometry

**DOI:** 10.1186/s12941-016-0129-1

**Published:** 2016-03-08

**Authors:** Laurène Deconinck, Agnès Meybeck, Maxime Pradier, Pierre Patoz, Hugues Melliez, Eric Senneville

**Affiliations:** Centre Hospitalier Dron, Service Universitaire des Maladies Infectieuses et du voyageur, 135 avenue du Président Coty, 59200 Tourcoing, France; Laboratoire de biologie, Centre Hospitalier Dron, 135 avenue du Président Coty, 59200 Tourcoing, France

**Keywords:** Fungemia, *Candida pulcherrima*, Mass spectrometry

## Abstract

**Background:**

Community-onset candidemia constitute a distinct clinical entity the incidence of which is increasing. Contribution of *non-albicans Candida* species is rising.

**Case presentation:**

We describe here the first reported case of community acquired fungemia due to *Candida pulcherrima*. Identification to the species level was performed by MALDI-TOF mass spectrometry. Treatment with fluconazole was successful.

**Conclusion:**

This case confirms the pathogenic role of *C. pulcherrima* and the contribution of MALDI-TOF mass spectrometry for identification of rare *Candida* species.

## Background

Candidemia is a major cause of morbidity and mortality in the health care setting [1]. Contribution of non-*albicans Candida* species to invasive infections is rising. Identification to the species level is essential for epidemiological investigations and optimal patient care [2]. *Candida pulcherrima* has been reported as potential pathogen [3]. To our knowledge, we report here for the first time a case of community acquired fungemia due to *C. pulcherrima*.

## Case presentation

### Observation

A 48-year-old man was admitted to our hospital because of fever, dyspnea, and chest pain. The patient had a history of hepatitis C virus infection, opioid-use disorder treated with buprenorphine, chronic venous insufficiency, and severe chronic respiratory failure due to chronic obstructive pulmonary disease treated with long-term oxygen therapy and non invasive ventilation at home. He was not admitted to the hospital and did not use any healthcare facilities in the last 12 month period. He reported recent use of cotton balls soaked in buprenorphine for injection in his venous ulcers.

On examination, the patient was noted to be short of breath with a respiratory rate of 24/min, and an oxygen saturation of 70 % on room air. The temperature was 39 °C, and the blood pressure 90/50 mmHg. There were scattered focal crackles in the right lung. The heart sounds were normal. Skin examination found chronic venous ulcers on both legs, no marks consistent with intravenous injection, no abscesses. Oral and dental health examination was normal. The remainder of the examination was normal. Arterial blood gas analysis revealed a PaO_2_ of 154 mmHg, a PaCO_2_ of 90 mmHg, a pH of 7.18 on 12 L/min of oxygen. The patient was admitted to the intensive care unit and placed under non-invasive ventilation. Blood cultures were taken before intravenous cefotaxime and gentamicine were started. Laboratory results disclosed the following: hemoglobin, 12.0 g/dL; white blood cells, 5710/mm^3^; platelets, 94,000/mm^3^; creatinine, 13 mg/L; blood urea nitrogen, 0.49 g/L; C-reactive protein, 89 mg/L; aspartate aminotransferase, 37 units/L; alanine aminotransferase, 16 units/L; total bilirubin, 5 mg/L. HIV serologic test and hepatitis C virus RNA quantitative PCR were negative. Chest X-ray showed distension with no infiltrate. Computed tomography of the chest revealed consolidation of the left upper lobe. The patient’s condition improved rapidly allowing his transfer to the department of infectious diseases. Two sets of blood cultures taken at admission grew yeasts on hospital day 2. Caspofungin 70 mg once a day was started. Identification to the species level was performed by Matrix-assisted laser desorption/ionization time-of-flight mass spectrometry (MALDI-TOF mass spectrometry) (Vitek MS, BioMerieux^®^, Marcy l’Etoile, France). A portion of one colony isolated from a Sabouraud agar plate (BioMérieux^®^, Marcy l’Etoile, France) was applied directly onto the Vitek MS disposable target (single deposit) and was lysed with 0.5 μl of 25 % formic acid. After drying completely at room temperature (1–2 min), 1 μL of ready-to-use α-cyano-4-hydroxycinnamic acid (CHCA) matrix (BioMérieux^®^, Marcy l’Etoile, France) was applied to the spot, which was allowed to dry completely again (1 min). Final identification result led to the diagnosis of *C. pulcherrima* blood-stream infection. Antifungal drug susceptibility testing was performed by E-test (BioMérieux^®^, Marcy l’Etoile, France). In vitro minimal inhibitory concentrations for fluconazole, voriconazole, caspofungin, and amphotericin B were: 0.25, 0.004, 0.25, and 0.047 mg/L respectively. Culturing of the patient’s injection drug apparatus could not be obtained.

Trans-oesophageal echocardiogram revealed no valvular abnormalities or vegetations. Optic fundus examination was normal. Abdominal computed tomography revealed no deep abcess. Yeasts were eradicated at the 2nd day of the antifungal therapy. Treatment was modified for fluconazole 400 mg once a day, according to the pathogen’s susceptibility pattern. The patient completed 2 weeks of antifungal therapy and recovered without complications.

## Discussion

We report a case of community-onset fungemia caused by *C. pulcherrima* occuring in an injection-drug user. Identification to the species level was performed by MALDI-TOF mass spectrometry.

*Candida pulcherrima* is part of the oral cavity flora in humans [4]. It might become an opportunistic pathogen [5]. Rare cases of fungemia have been reported. All previous cases of *C.pulcherrima* blood-stream infections have occured in healthcare setting and were related to the use of indwelling catheter for parenteral nutrition [6–8]. We reported for the first time a case of *C.pulcherrima* causing community acquired fungemia. Community-onset candidemia constitute a distinct clinical entity the incidence of which is increasing [9]. Signs and symptoms of candidemia are non-specific leading to diagnostic delay. In our patient, diagnosis of fungemia was not initially suspected despite recent history of intravenous drug use and antifungal therapy was not included in the first empiric therapy regimen. Twenty-four hour after admission, blood cultures grew yeasts and use of MALDI-TOF mass spectrometry conducted to rapid diagnosis of *C. pulcherrima* blood-stream infection. Injection drug users are at increased risk for blood-stream fungal infections. Pathogenesis involves mycotic contamination of drug paraphernalia (e.g., used syringes) or contaminated drug solutions. Injected-buprenorphine users usually crush tablets in water or saliva and filter the solution through cotton-pad. Severe infectious complications have been reported following buprenorphine injections, including fungal systemic infection [10]. In our case, culturing of the patient’s injection drug apparatus and of the cotton ball could not be obtained.

Although reported cases of fungemia due to *C.pulcherrima* are few, identification of *C.pulcherrima* is difficult, and might lead to underestimation of its incidence and pathogenic role. Morphology and physiology of *C.pulcherrima* are very close to those of *C.lusitaniae* [11]. No phenotypic test can discriminate definitely between these two species. Molecular methods such as PCR are more reliable than conventional laboratory methods for identification of fungal pathogens. But these methods are not affordable in many laboratories.

Several investigators have demonstrated that MALDI-TOF mass spectrometry could accurately identify yeasts [12, 13]. This technique is based upon the acquisition by the desorption of specific proteins or glucans from fungal cells of unique mass spectrometric profiles (fingerprints).

Rapid recognition of candidemia and prompt initiation of appropriate antifungal therapy is a key determinant of outcome. For certain species, susceptibilities to antifungal agents can be predicted based on epidemiological susceptibility data. Concerning *C. pulcherrima*, available data are limited and reveal in vitro susceptibility to fluconazole [14, 15]. Clinical breakpoints have not yet been established for echinocandins, amphotericin B, and voriconazole by the Clinical and Laboratory Standards Institute (CLSI) Subcommittee on Antifungal Susceptibility Testing and the European Committee on Antimicrobial Susceptibility Testing (EUCAST) [16]. In our case, results of antifungal drug susceptibility testing were concordant with previously published data, and treatment with caspofugin, rapidly switched for fluconazole was successful.

## Conclusions

This case confirms the pathogenic role of *C. pulcherrima* and illustrates the contribution of MALDI-TOF mass spectrometry for correct identification of rare *Candida* species. Prompt identification to the species level can predict in vitro susceptibility and ensuring early appropriate therapy.

## Consent

Written informed consent was obtained from the patient for publication of this case report.

## References

[1] Montagna MT, Caggiano G, Lovero G, De Giglio O, Coretti C, Cuna T (2013). Epidemiology of invasive fungal infections in the intensive care unit: results of a multicenter italian survey (AURORA Project). Infection.

[2] Horn DL, Neofytos D, Anaissie EJ, Fishman JA, Steinbach WJ, Olyaei AJ (2009). Epidemiology and outcomes of candidemia in 2019 patients: data from the prospective antifungal therapy alliance registry. Clin Infect Dis.

[3] Hazen KC (1995). New and emerging yeast pathogens. Differentiation between atypical isolates of *Candida lusitaniae* and *Candida pulcherrima* by determination of mating yype. Clin Microbiol Rev.

[4] Sahand IH, Maza JL, Eraso E, Montejo M, Aguirre JM, Quindos G (2009). Evaluation of CHROM-Pal medium for the isolation and direct identification of *Candida dubliniensis* in primary cultures from the oral cavity. J Med Microbiol.

[5] Pospisil L (1989). The significance of *Candida pulcherrima* findings in human clinical specimens. Mycoses.

[6] Weber A, Kolb S (1986). The repeated isolation of *Candida pulcherrima* (Lindner) Windisch from blood cultures of a patient on parenteral nutrition. Mykosen.

[7] Mohl W, Lerch MM, Klotz M, Freidank H, Zeitz M (1998). Infection of an intravenous port system with *Metschnikowia pulcherrima* Pitt et Miller. Mycoses.

[8] Bereczki L, Bartha N, Kocsubé S, Soki J, Lengyel G, Talosi G (2012). Fungaemia caused by *Candida pulcherrima*. Med Mycol.

[9] Pfaller MA, Moet GJ, Messer SA, Jones RN, Castanheira M (2011). Candida bloodstream infections: comparison of species distributions and antifungal resistance patterns in community-onset and nosocomial isolates in the SENTRY Antimicrobial Surveillance Program, 2008–2009. Antimicrob Agents Chemother.

[10] Grau D, Vidal N, Faucherre V, Léglise Y, Pinzani V, Blayac JP (2010). Infectious adverse events related to misuse of high-dose buprenorphine: a retrospective study of 42 cases. Rev Med Interne.

[11] Noel T, Favel A, Michel-Nguyen A, Goumar A, Fallague K, Chastin C (2005). Differentiation between atypical isolates of *Candida lusitaniae* and *Candida pulcherrima* by determination of mating type. J Clin Microbiol.

[12] Taj-Aldeen SJ, Kolecka A, Boesten R, Alolaqi A, Almaslamani M, Chandra P (2014). Epidemiology of candidemia in Qatar, the Middle East: performance of MALDI-TOF MS for the identification of *Candida* species, species distribution, outcome, and susceptibility pattern. Infection.

[13] Chao QT, Lee TF, Teng SH, Peng LY, Chen PH, Teng LJ (2014). Comparison of the accuracy of two conventional phenotypic methods and two Maldi-Tof MS systems with that of DNA sequencing analysis for correctly identifying clinically encountered yeasts. PLoS ONE.

[14] Mandras N, Tullio V, Allizond V, Scalas D, Banche G, Roana J (2009). In vitro activities of fluconazole and voriconazole against clinical isolates of *Candida* spp. Determined by disk diffusion testing in Turin, Italy. Antimicrob Agents Chemother.

[15] Messer SA, Kirby JT, Sader HS, Fritsche TR, Jones RN (2004). Initial results from a longitudinal international surveillance programme for anidulafungin. J Antimicrob Chemother.

[16] Pfaller MA, Castanheira M, Diekema DJ, Messer SA, Moet GJ, Jones RN (2010). Comparison of European Committee on Antimicrobial Susceptibility Testing (EUCAST) and Etest methods with the CLSI broth microdilution method for echinocandin susceptibility testing of *Candida* species. J Clin Microbiol.

